# A comparative study of bibliometric analysis on old adults’ cognitive impairment based on Web of Science and CNKI via CiteSpace

**DOI:** 10.1186/s13561-023-00470-7

**Published:** 2023-12-02

**Authors:** Shuyi Yan, Mingli Pang, Jieru Wang, Rui Chen, Hui Liu, Xixing Xu, Bingsong Li, Qinling Li, Fanlei Kong

**Affiliations:** 1 Centre for Health Management and Policy Research, School of Public Health, Cheeloo College of Medicine, Shandong University, Jinan, 250012 China; 2 NHC Key Lab of Health Economics and Policy Research, Shandong University, Jinan, 250012 China; 3 Institute of Health and Elderly Care, Shandong University, Jinan, China

**Keywords:** Cognitive impairment, Old adults, Alzheimer's disease, Bibliometrics, Visualization analysis, CiteSpace

## Abstract

**Introduction:**

The purpose of this study was to analyze the current status, the research hot spots and frontiers of cognitive impairment (CI) on old adults from 2012 to 2022 based on Web of Science (WoS) and China National Knowledge Infrastructure (CNKI) via CiteSpace, and provide new in-sights for researchers.

**Methods:**

The articles regarding the old adults’ CI in the WoS and CNKI were retrieved from 2012 to 2022. CiteSpaceV.6.1.R4 was used to generate network maps.

**Results:**

Four thousand seven hundred thirteen publications and 304 publications from CNKI were retrieved. Overall, from 2012 to 2022, the trend of articles published in WoS and CNKI were increasing. Data from WoS showed that USA, University of California, Petersen RC were the most influential country, institution and author respectively; Folstein MF, Neurology and a diagnosis guideline of mild CI were the most cited author, journal and reference separately; while the keywords of CI could be summarized in 3 aspects: related disease and symptom, risk factors, manifestations. Data from CNKI illustrated that Peking Union Medical College, Dan Liu were the most influential institution and scholar respectively, while the keywords of CI could be summarized in 3 aspects: related disease and symptoms, risk factors, intervention.

**Conclusion:**

Articles published on old adults’ CI were drawing an increasing amount of attention from 2012 to 2022 both in WoS and CNKI. Keywords of CI in WoS and CNKI both focused on risk factors, related disease and symptom, yet WoS contributed more to the mechanism and CNKI contributed more to the intervention.

## Background

Population aging has become a public health issue [1], and the size of population aged 60 or older is predicted to rise to 1.4 billion, 2.1 billion, and eventually 3.1 billion people by 2030, 2050, and 2100, respectively [2]. As for China, the number of Chinese elderly people over the age of 60 had reached 254 million in 2020, accounting for 18.7% of the total population [3], which is estimated to increase to 34.6% in 2050 [4]. More and more old adults indicated an increased burden of age-related diseases, such as cognitive impairment (CI) [4]. Cognitive impairment is a kind of common age-related diseases, which is a deficit in one or more key brain functions (such as memory, learning, concentration, and decision making) that ranges from mild to severe, with severe impairment that impairs daily living and independence [5].

Overall, as the population ageing, the incidence of cognitive impairment also increased, which furtherly made the cognitive impairment of the elderly received more concern and summarizing previous researches and updating the current frontiers of this field were imperative [6, 7]. However, most existed articles have focused on specific types of cognitive impairment [8–10], yet few have concentrated on the current general research proceedings of this field. Thus, this study aimed to clarify the research trend and hotspots of old adults’ CI from 2012 to 2022 and furtherly assist scholars worldwide in better understanding the current situation and frontiers by using Citespace.

## Materials and methods

### Materials

Bibliometric analysis relies on literature database. The data sources for this study include the Web of Science (WoS) and China National Knowledge Infrastructure (CNKI). The data collection procedure for the current study is presented in. Web of Science database contains large multidisciplinary, high-impact, international, comprehensive academic journals, which is a relatively comprehensive citation database, ensuring the representativeness and authority of literature sources [11]. Considering that it has the highest applicability to CiteSpace software, it was selected as the preferred retrieval database in this study [12]. CNKI is the largest and key Chinese-language literature database in China, covering more than 99% of Chinese academic and practical journals, which ensures the representativeness and authority of the literature sources [13], which was also chosen as the retrieval database in this study.

**Fig. 1:**
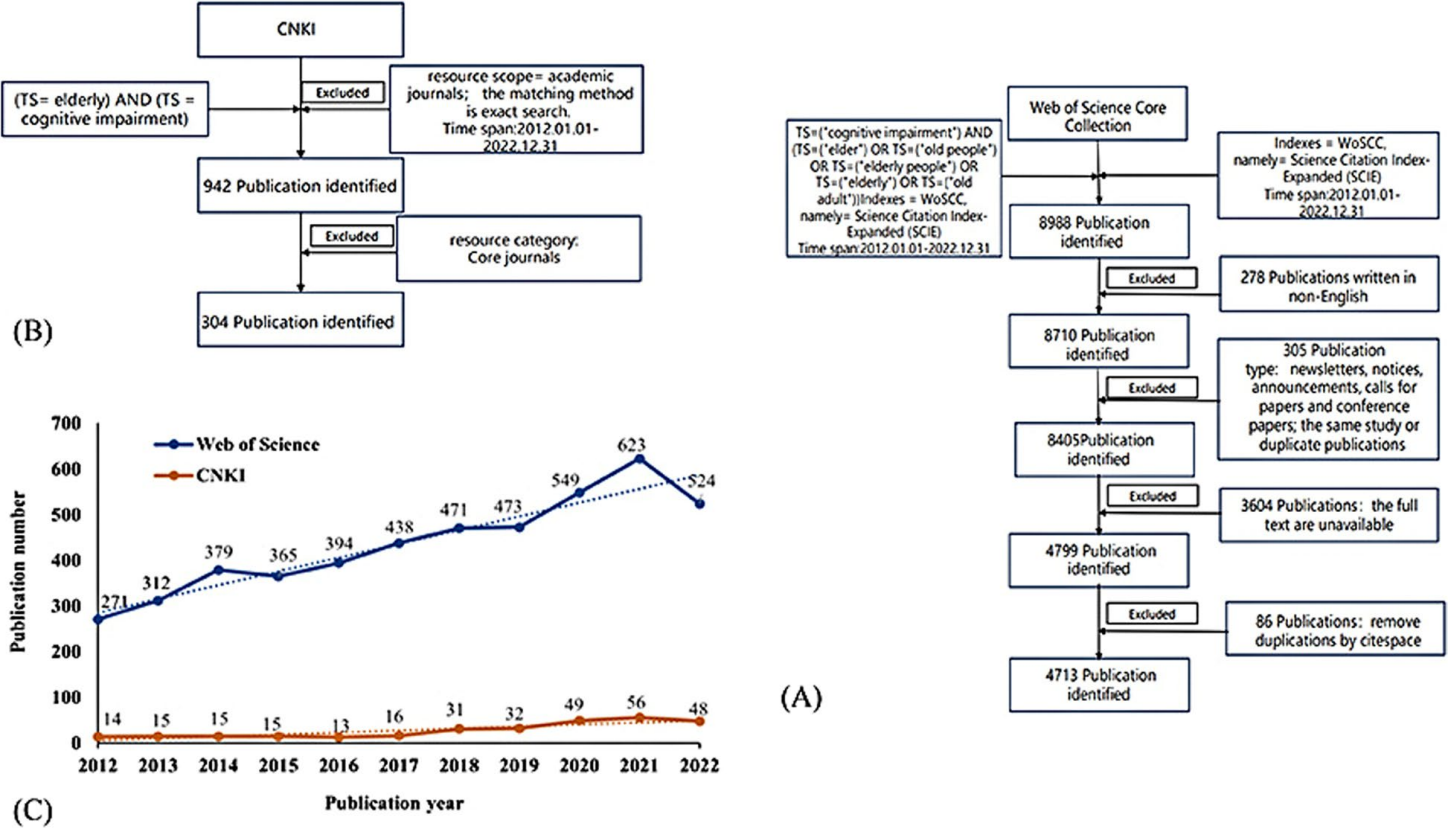
**A** Data collection procedure in WoS. **B** Data collection procedure in CNKI. **C** The annual number of publications on old adults’ CI in WoS and CNKI from 2012–2022

The authors extracted and downloaded relevant publications from the Web of Science (WoS) on December 4th 2022, and used the following search strategy: TS = ("cognitive impairment") AND (TS = (“elder”) OR TS = (“old people”) OR TS = (“elderly people”) OR TS = (“old adult”) OR TS = (“elderly”)); Indexes = Web of Science Core Collection (WoSCC); namely = Science Citation Index-Expanded (SCIE); Language = English; publication time from 2012.1.1 to 2022.12.9. There were no limitations placed on the type of study reviewed. The search excluded the following: newsletters, notices, announcements, calls for papers and conference papers; the same study or duplicate publications; literature with incomplete data and literature where the full text was unavailable [14]. Ultimately, we identified 4713 articles and reviews for visual analysis. The data collection procedure is shown in Fig. 1(A).

Data from the China National Knowledge Infrastructure (CNKI) were extracted on December 4th 2022 and downloaded on the same day. The search strategy was as follows: TS = ("cognitive impairment") AND ((TS = ("elder") OR TS = ("old people") OR TS = ("elderly people") OR TS = (“old adult”) OR TS = ("elderly")); publication time from 2012.1.1 to 2022.12.9; Select academic journals, data sources are Core Journals. Ultimately, we identified 304 articles and reviews for visual analysis. The data collection procedure is shown in Fig. 1(B).

### Methods

CiteSpace is a bibliometric analysis visualization software developed by Professor Chaomei Chen, which uses the Java platform and is an interactive analysis tool that uses a combination of bibliometrics, visual analysis methods and data mining algorithms [15]. CiteSpace provides a variety of functional options for bibliometric research, which includes collaborative network analysis, co-occurrence analysis and co-citation analysis. Through these the researchers could explore the current research status, research hotspots, research frontiers and evolution of a scientific field by generating a series of visual knowledge maps, revealing the research directions, research stages and frontier characteristics of institutions and authors, and ultimately determining the trend of the field [12].

The visualization knowledge network created by CiteSpace consisted of nodes and lines. The nodes in the network stood for items, such as countries, institutions, authors, cited authors, keywords and cited references, and lines between the nodes represented cooperation, co-occurrence, or co-citation relationships. The size of each node indicated the count. Each node was represented by a series of citation rings representing different years, and the thickness of the ring was proportional to the citation count in the corresponding time zone. Purple rings indicate that these countries/regions, institutes, or authors have greater centrality indicating hot spots or pivotal points in a field [16]. When the clustering function was initiated, it showed the frontiers of the research, the Modularity Q, and Mean Silhouette scoring which remarkably influenced visualization, reflecting the general structure feature of the net. Normally, Q > 0.3 denotes a generally significant structure. If S ≥ 0.5, the cluster will be deemed reasonable. Moreover, emerging trends could be tagged by using a citation burst [17]. This study utilized CiteSpace V.6.1.R4 to visually analyze the knowledge map of countries, institutions, authors, journals, references, and keywords concerning relevant research. Besides CiteSpace V.6.1.R4, Microsoft Office Excel 2021 was also used to manage data and analyze annual publications.

Before running the CiteSpace software, the parameters were set as follows: (1) a time span was chosen ranging from 2012 to 2022; (2) the year per slice was set as 1; (3) the node type was set as author/institution/keyword/reference/journal; (4) threshold selection criteria were set for the top 50, which means that data were extracted on the top 50 results for each time slice; (5) pruning was set as Path finder and Pruning sliced network. The remaining parameters were the default settings.

## Results

### The trend of publication outputs 

#### Data from WoS

Over the past 10 years, the number of annual publications from WoS has been increasing, as can be seen in Fig. 1(C). Specifically, there was a steadily increasing trend from 2013 to 2021, and the output reached a peak in 2021 with 623 publications, implying this is still a young field.

#### Data from CNKI

The number of articles from CNKI was less than WoS, but the general trend of articles published was on the rise. As shown in Fig. 1(C), before 2016, annual output of articles was stable. Since 2016, there was a rapid growth trend, which increased from 16 to 56. The number of publications in 2022 were slightly less than 2021, that may be due to the limitation of the search time, the articles have not been fully counted. As illustrated in Fig. 1(C), the old adults’ CI has received continuous attention and extensive research.

### Analysis of countries and institutions 

#### Data from WoS

**Fig. 2:**
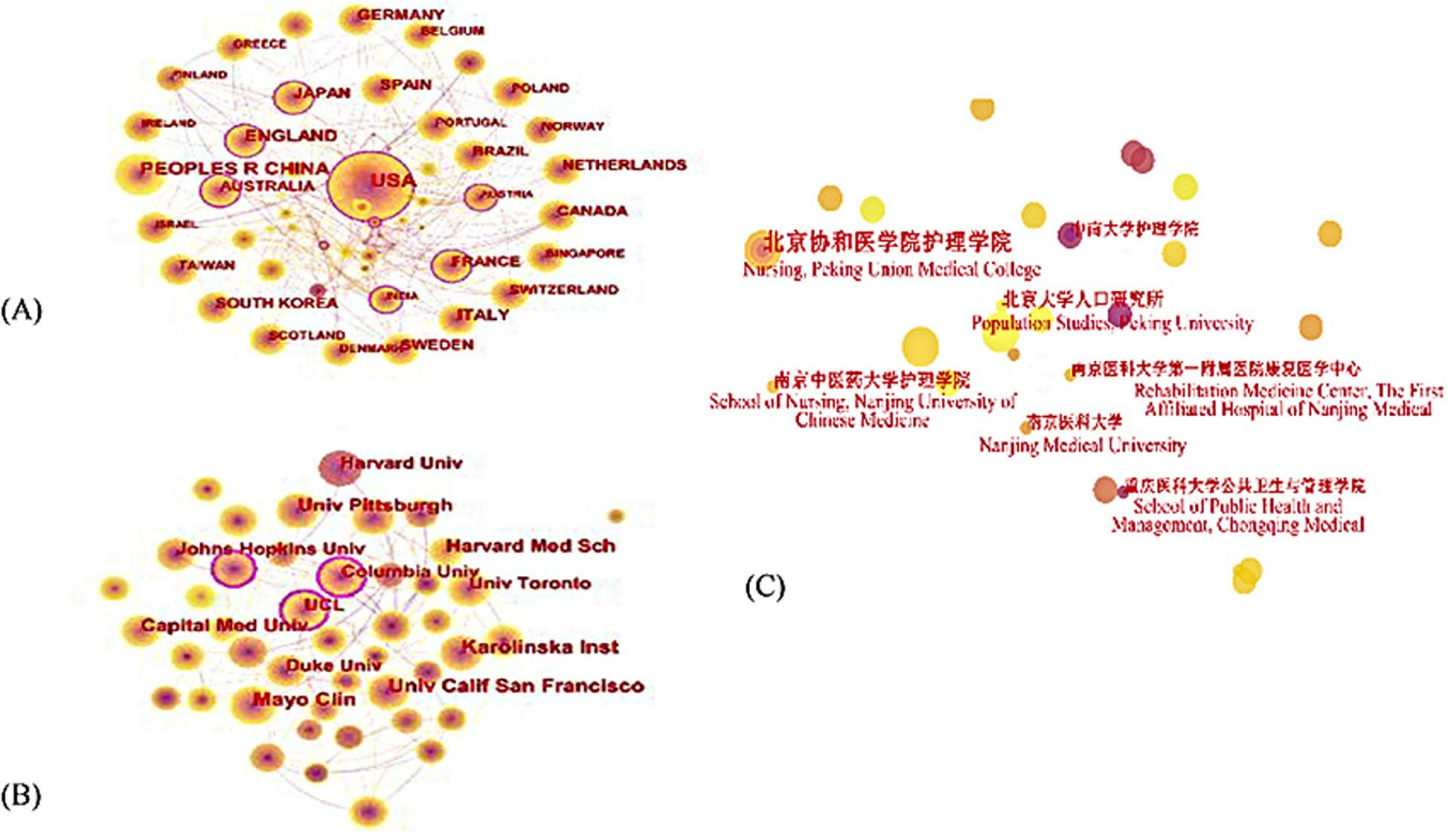
**A** Analysis of Countries on old adults’ CI in WoS. **B** Analysis of Institutions on old adults’ CI in WoS. **C** Analysis of Institutions on old adults’ CI in CNKI

Collaborative network between countries/regions was illustrated in Fig. 2(A), with 100 nodes and 486 link lines. The top ten countries in terms of number of publications and their centrality were listed in Table 1. The top five countries or regions in numbers of research articles were the USA (1456), China (827), England (454), Italy (368) and Japan (292). As for centrality, among the top 10 countries, France and India had the most centrality with 0.20 in the network. The centrality of England, Australia and USA were 0.17, 0.15 and 0.14, respectively, implying these countries played a strong role as a bridge in the cooperation between countries. Although China, Italy, Germany, Spain, Canada and Netherlands had published numerous papers, their centricity is less significant than 0.1, indicating they had less collaboration with other countries.

**Table 1:** Top 10 countries/regions on old adults’ CI in WoS

Ranking	Country/region	Frequency	Ranking	Country/region	Centrality
1	USA	1456	1	France	0.20
2	China	827	2	India	0.20
3	England	454	3	England	0.17
4	Italy	368	4	Australia	0.15
5	Japan	292	5	USA	0.14
6	Germany	271	6	Japan	0.13
7	Spain	254	7	Austria	0.11
8	France	242	8	Mexico	0.11
9	Canada	240	9	Pakistan	0.11
10	Netherlands	238	10	Spain	0.08

Collaborative network between institutions was illustrated in Fig. 2(B), with 478 nodes and 1395 link lines. The top 5 institutions contributing the most in this area were University of California (118), Karolinska Institute (94), Mayo Clinic (94), University of Pittsburgh (81), and Capital Medical University (79). American's contribution to this field was the most outstanding, 60% of institutions came from them. Except for Karolinska Institute in Sweden, Capital Medical University in China, University College London in England and University of Toronto in Canada, the rest Institutions were all located in the United States. Among these institutions, Harvard Medical School, Columbia University and Johns Hopkins University have a high centrality, showing these institutions had a higher influence and cooperation. All these could be seen in Table 2.

**Table 2:** Top 10 institutions on old adults’ CI in WoS

Ranking	Institution	Frequency	Ranking	Institution	Centrality
1	University of California, San Francisco	118	1	Columbia University	0.14
2	Karolinska Institute	94	2	University College London	0.11
3	Mayo Clinic	94	3	University of Michigan	0.09
4	University of Pittsburgh	81	4	Duke University	0.09
5	Capital Medical University	79	5	University of California, Los Angeles	0.08
6	Harvard Medical School	73	6	King's College London	0.08
7	University College London	66	7	Leiden University	0.08
8	University of Toronto	64	8	Capital Medical University	0.08
9	Columbia University	64	9	The University of Melbourne	0.08
10	Johns Hopkins University	63	10	Sungkyunkwan University	0.08

#### Data from CNKI

Figure 2(C) consists of 242 nodes and 164 links. The connection between institutions was sparse in China, since 242 institutions were included in the network while 196 (80.99%) published only one article. The institutions with the highest number of articles published was Peking Union Medical College (9), followed by the Institute of Peking University (5), Nanjing University of Chinese Medicine (5), Meanwhile, the centrality of all these institutions were 0, indicating the cooperation and communication between these institutions were infrequent. All these could be seen in Table 3.

**Table 3:** Top 10 Institutions/Author on old adults’ CI in CNKI

Ranking	Institution	Frequency	Centrality	Author	Frequency	Centrality
1	Peking Union Medical College	9	0.00	Dan Liu	6	0.00
2	Peking University	5	0.00	Yan Zeng	5	0.00
3	Nanjing University of Chinese Medicine	5	0.00	Guirong Cheng	5	0.00
4	Chongqing Medical University	4	0.00	Zheng Li	5	0.00
5	The First Affiliated Hospital of Nanjing Medical University	4	0.00	Han Wu	4	0.00
6	Nanjing Medical University	4	0.00	Hui Zeng	4	0.00
7	Central South University	4	0.00	Yi Zhu	3	0.00
8	Chinese Center for Disease Control and Prevention	3	0.00	Taixia Song	3	0.00
9	Fujian University of Chinese Medicine	3	0.00	Yingjie Li	3	0.00
10	Gulou Hospital of Nanjing University Medical School	3	0.00	Hongmei Yu	3	0.00

### Analysis of authors and co-cited authors

#### Data from WoS

**Fig. 3:**
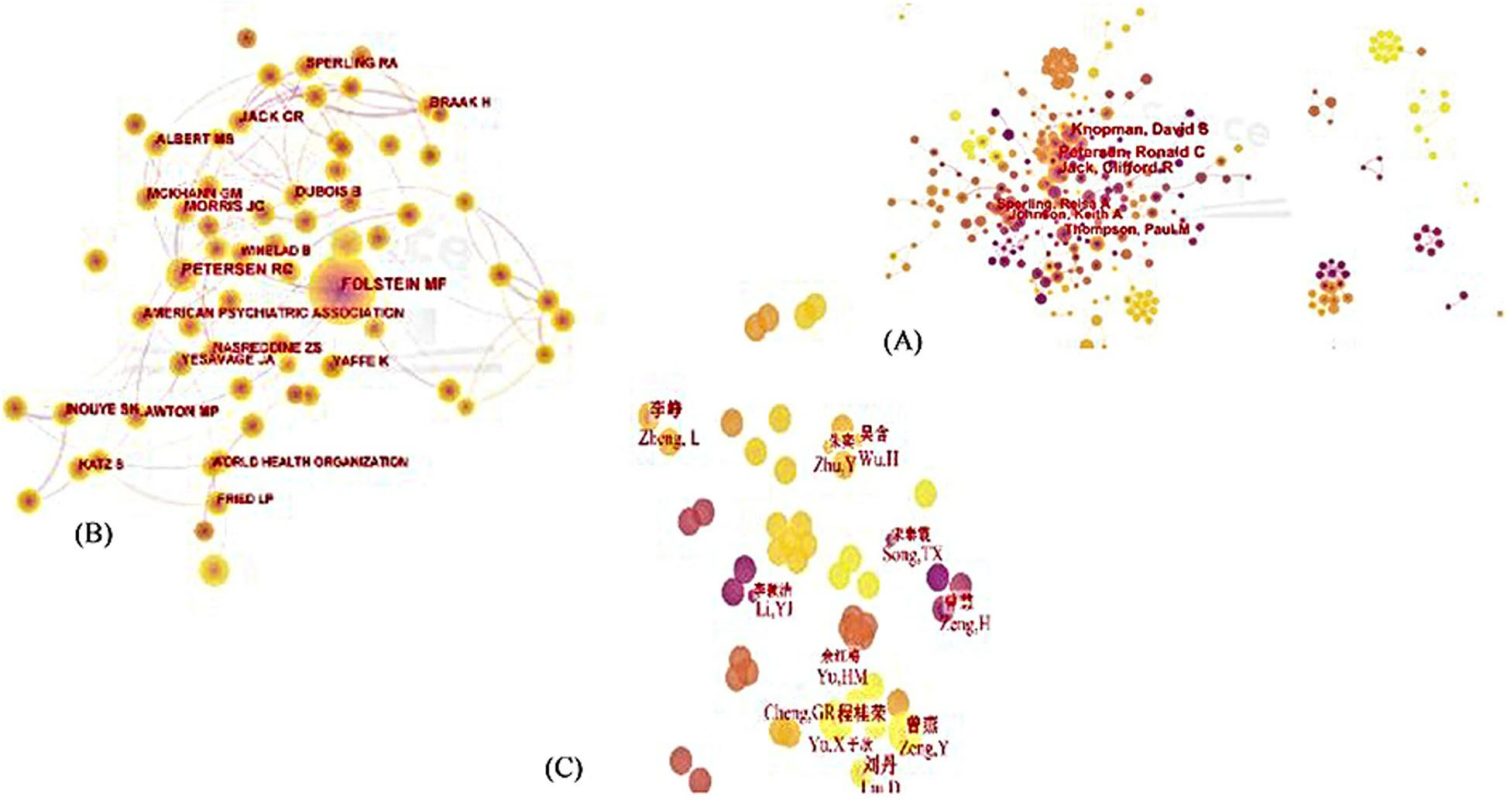
**A** Analysis of authors old adults’ CI in WoS. **B** Analysis of cited authors old adults’ CI in WoS. **C** Analysis of authors on old adults’ CI in CNKI

Collaborative network between authors was illustrated in Fig. 3(A), with 585 nodes and 1122 link lines. Concerning the number of publications, Petersen RC was the most prolific author with 38 articles, followed by Jack CR (35), Knopman DS (34), Thompson PM (25) and Johnson KA (22), as shown in Table 4. Many stable academic groups had formed, and academic groups located at the center of the network tended to appear earlier, while those that appeared later were distributed around the network map. Meanwhile, it was worth noting that all these authors had a centrality less than 0.1, suggesting that the cooperation and communication between these top authors were infrequent. In the network of co-authors, each node represents one author, and the size of the node indicates the number of publications of the author. A larger node means more publications. Figure 3(B) consisted 949 nodes and 2872 link lines and most frequently cited was Folstein MF with 1063, followed by Petersen RC (888), Jack CR (479), Morris JC (466), and Albert MS (350).

**Table 4:** Top 10 authors on old adults’ CI in WoS

Ranking	Author	Frequency	Ranking	Author	Centrality
1	Petersen, RC	38	1	Ewers, M	0.08
2	Jack, CR	35	2	Thompson, PM	0.06
3	Knopman, DS	34	3	Jack, CR	0.05
4	Thompson, PM	25	4	Knopman, DS	0.05
5	Johnson, KA	22	5	Bennett, DA	0.05
6	Sperling, RA	21	6	Weiner, MW	0.04
7	Yaffe, Kristine	20	7	Blennow, K	0.04
8	Boeve, BF	20	8	Chetelat, G	0.04
9	Roberts, RO	19	9	Vellas, B	0.04
10	Blennow, K	19	10	Petersen, RC	0.03

#### Data from CNKI

Collaborative network between authors was illustrated in Fig. 3(C), with 328 nodes and 328 link lines. The number of articles published by these authors was less than in WoS, and 238 (72.56%) published only one article. For a better interpretation, profiles of associated scholars were exhibited in Table 3. Dan Liu was the most active author, with 6 publications, followed by Yan Zeng (5), Guirong Chen (5), Zheng Li (5) and Han Wu (4); the centrality of all these authors were 0.

### Analysis of keywords

#### Data from WoS

**Fig. 4:**
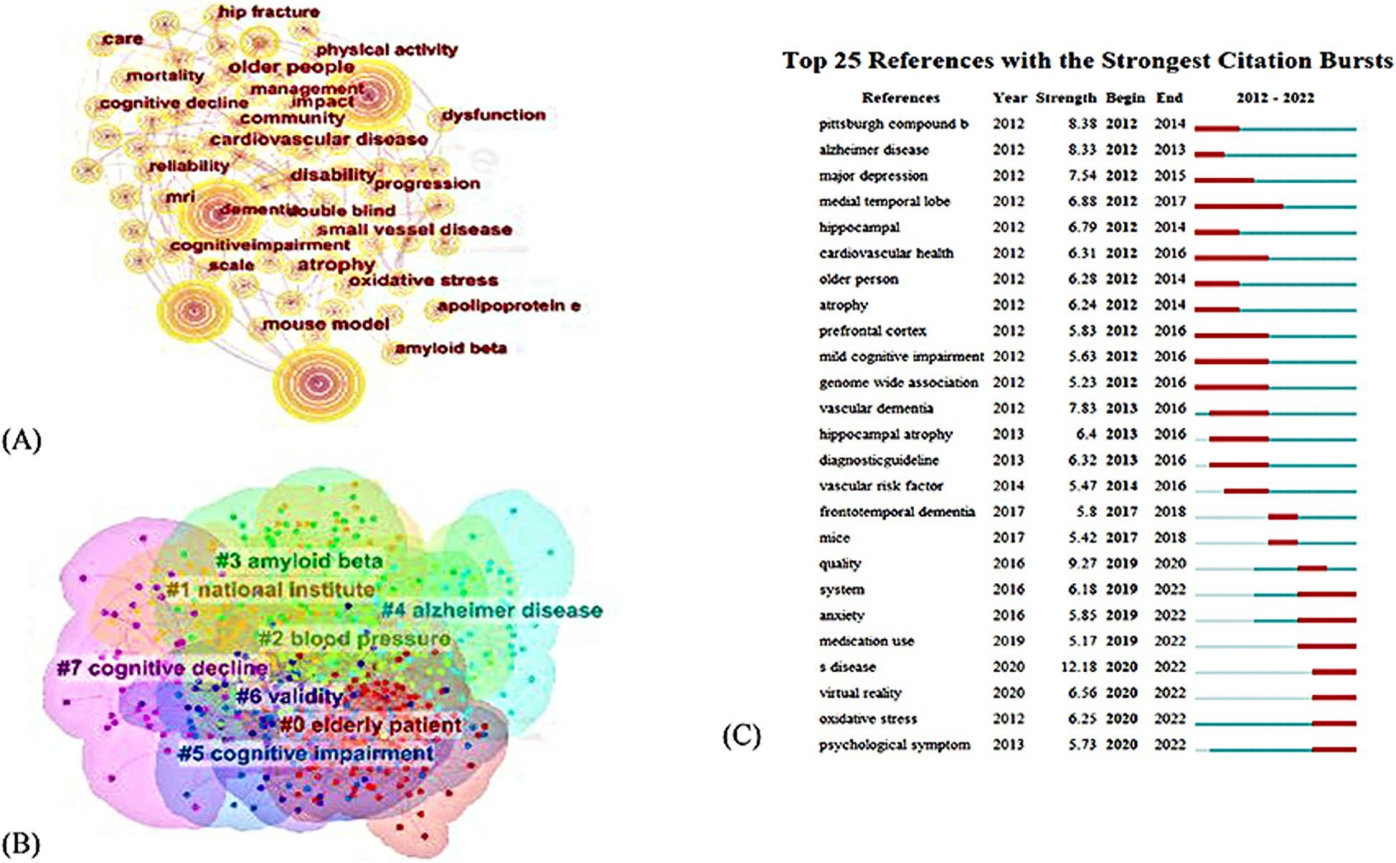
**A** Analysis of keywords on old adults’ CI in WoS. **B** Analysis of keywords clustering on old adults’ CI in WoS. **C** Top 25 keywords with strongest citation bursts in WoS

The co-occurring keywords network, as shown in Fig. 4(A), consisted of 686 nodes and 3388 collaboration lines. Table 5 presents the top 10 keywords with high frequency. After removing the major search terms, it was found that “Alzheimer’s disease” was the most popular keyword with 1666, followed by “dementia”, “mild cognitive impairment”, “risk” and “prevalence”. Accordingly, the keywords of CI could be summarized in 3 aspects: 1) the related disease and symptoms of old adults’ CI; 2) the risk factors of old adults’ CI; 3) the biological mechanism of occurrence of old adults’ CI.

**Table 5:** Top 10 keywords on old adults’ CI in WoS and CNKI

Ranking	Frequency	Keywords	Ranking	Frequency	Keywords
1	1666	alzheimers disease	1	33	cognitive function
2	1298	dementia	2	23	influencing factors
3	1185	mild cognitive impairment	3	17	review
4	604	risk	4	17	depression
5	476	prevalence	5	14	risk factors
6	464	elderly patient	6	12	dementia
7	432	impairment	7	11	prevalence
8	409	decline	8	9	fragility
9	402	association	9	8	Nursing Home
10	311	disease	10	8	screening

All the keywords in this study were classified into 8 clusters (Table 6) after the clustering analysis of high-frequency keywords (more than 100 times) was performed. The visualization map obtained nodes were 686, links were 3388, the Modularity Q score was 0.4017, the Mean Silhouette score was 0.7178, implying the clustering was reasonable and structurally significant, as presented in Fig. 4(B). Three main topics highlighted by different keywords were categorized as follow: 1) related diseases of old adults’ CI (e.g., #1 diagnostic guideline #4 Alzheimer’s disease, #5 cognitive impairment, #7 cognitive decline); 2) risk factors of old adults’ CI (e.g., #0 elderly patient, #2 blood pressure); 3) the mechanism of old adults’ CI (e.g., #3 amyloid beta, #6 validity).

**Table 6:** Summary of the largest nine clusters in WoS

Cluster	Size	Year	Lable(LLR)
0	127	2014	elderly patient; alzheimers disease; hip fracture; mild cognitive impairment; older people
1	120	2015	national institute; mild cognitive impairment; diagnostic guideline; functional connectivity; cognitive impairment
2	104	2014	blood pressure; hypertension; small vessel disease; magnetic resonance imaging; white matter hyperintensities
3	86	2015	amyloid beta; oxidative stress; tau; alzheimers disease; neuroinflammation
4	67	2017	alzheimer disease; cognitive dysfunction; neurovascular coupling; propofol; anesthesia
5	66	2015	cognitive impairment; elderly; dementia; physical activity; prevalence
6	64	2015	validity; validation; reliability; mild cognitive impairment; prediction
7	50	2018	cognitive decline; cerebral small vessel disease; systematic review; machine learning; healthy aging

The top 25 keywords with the strongest occurrence burst were shown in Fig. 4(C). Keywords “s disease” with the strongest citation bursts appeared in 2020, followed by “Pittsburgh compound b” (8.38), “alzheimer disease” (8.33). Medial temporal lobe (2012–2017), cardiovascular health (2012–2016), mild cognitive impairment (2012–2016), genome wide association (2012–2016) had been studied for a long time, representing they had been a hot topic for some time. The keywords with occurrence burst lasting until 2022 represent hot topics, “system anxiety”, “medication use”, “s disease”, “virtual reality”, “oxidative stress” and “psychological symptom” would be potentially cited frequently over the coming years.

#### Data from CNKI

**Fig. 5:**
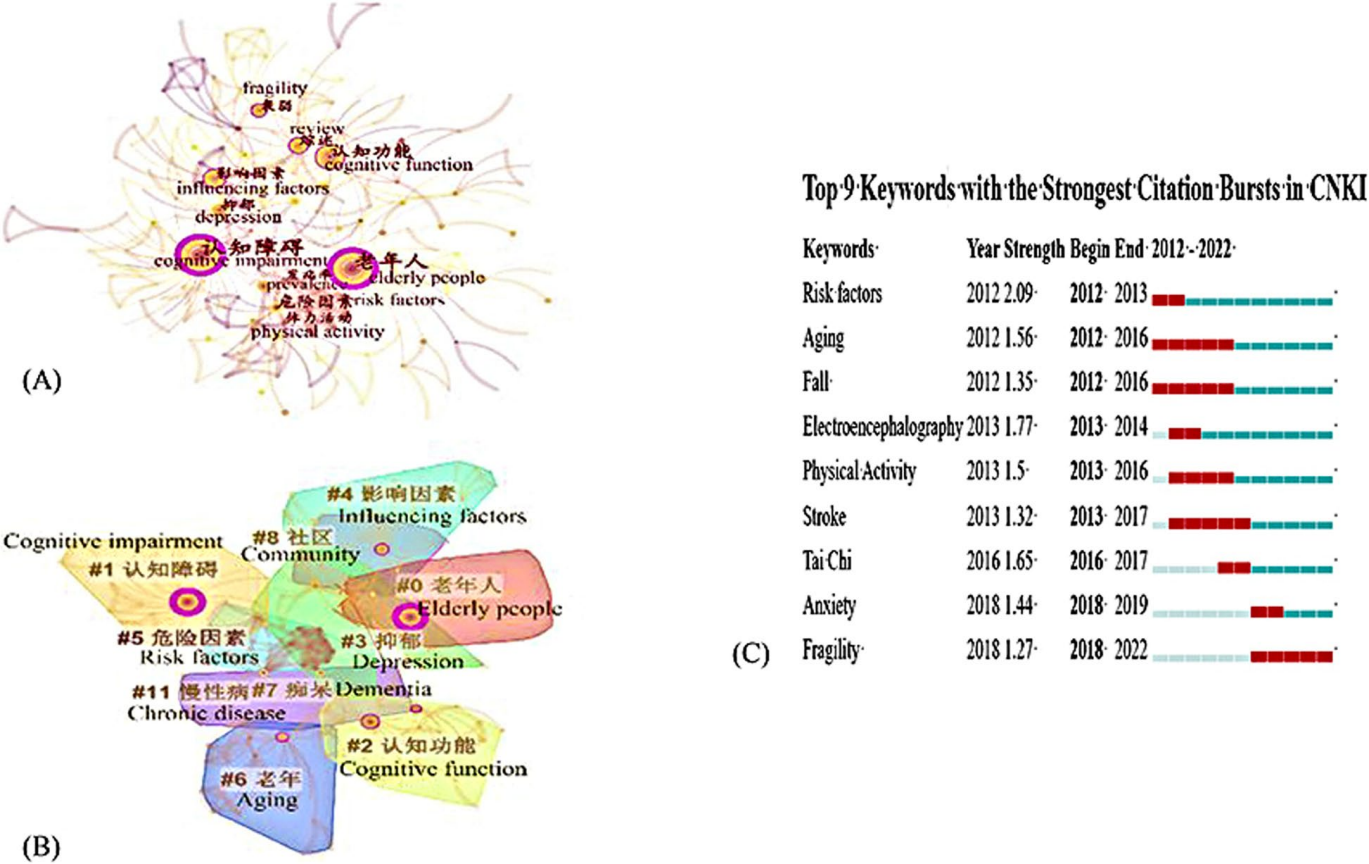
**A** Analysis of keywords on old adults’ CI in CNKI. **B** Analysis of keywords clustering on old adults’ CI in CNKI. **C** Top 9 Keywords with the Strongest Citation Bursts in CNKI

Figure 5(A) indicated that 280 nodes and 521 links composed of the merged co-occurring keywords network. Table 5 presented the top 10 keywords with high frequency. After removing major search terms, the maximum frequency was of “cognitive function” at 33, followed by “influencing factors”, “review”, “depression” and “risk factors” with 23, 17, 17, 14 respectively. According to the above keywords, the research hotspots of old adults’ CI could be summarized in 3 aspects: 1) diseases and symptoms associated with old adults’ CI; 2) the risk factors of old adults’ CI; 3) interventions.

The visualization map obtained nodes were 705, links were 3375, the Modularity Q score was 0.6543, the Mean Silhouette score was 0.8953, indicating the clustering was reasonable and structurally significant. Figure 5(B) showed a cluster visualization of the keyword co-citation network which was divided into ten co-citation clusters. Moreover, three main topics highlighted by different keywords could be categorized into 3 kinds as follow: 1) related disease and symptoms of CI (#2 cognitive function, #7 dementia, #11 chronic disease); 2) the risk factors that contribute to cognitive decline (#0 elderly people, #3 depression, #4 influencing factors, #5 risk factors; #6 aging,); 3) intervention of CI (#1 screening#8 community). The summary of the largest nine clusters was shown in Table 7.

**Table 7:** Summary of the largest nine clusters in CNKI

Cluster	Year	Label (LLR)
0	2018	Elderly people; Dual task; EEG; Depression; Cognitive function
1	2017	Cognitive impairment; Screening; Diabetes; Mild cognitive impairment; Mortality risk
2	2018	Cognitive function; Tai Chi; Executive function; Weakness; Memory
3	2018	Depression; nursing home; anxiety; sleep disorders; intellectual activity
4	2018	Influencing factors; Middle-aged and elderly; Rural; Prevalence; Amnestic mild cognitive impairment
5	2015	Risk factors; Cognition; Physical activity; Genetic risk; Hypotension
6	2014	Geriatric; Falls; Review; aging; Stroke
7	2017	Dementia; Early intervention; Prevalence; Marital status; Early identification
8	2018	Community; WeChat use; Circadian rhythm; Blood pressure; Cognitive function
11	2016	Chronic disease; Empty nest; Smoking; Mild cognitive impairment; Older adults

Top 9 keywords with strongest citation bursts were presented in Fig. 5(C). The blue line indicated the time interval, while the red line indicates the time period when a keyword had a burst. Keyword “risk factors” with the strongest bursts appeared in 2012, while aging (2012–2016), fall (2012–2016), stroke (2013–2017) had been studied for a long time, indicating that scientists had invested a lot of time and funding in these directions. Keyword “fragile” appeared in 2018 and continued to present, indicating that Chinese scholars were focusing on this filed.

### Analysis of cited reference

The top five cited references about frequency were shown in Table 8. The first ranked citation was the article published in Alzheimer’s & Dementia, titled “The diagnosis of mild cognitive impairment due to Alzheimer's disease: Recommendations from the National Institute on Aging-Alzheimer's Association workgroups on diagnostic guidelines for Alzheimer's disease”, which developed criteria for the mild cognitive impairment [18]. Besides, three of the top five reference involved in the diagnostic guidelines of CI. Most of the top five citations were all published in the TOP journals: four were published in Alzheimers & Dementia [Impact factor = 16.655], and one was published in Lancet [Impact factor = 202.731].

**Table 8:** The top five cited references in WoS

Ranking	Author	Title	Journal
1	Albert MS	The diagnosis of mild cognitive impairment due to Alzheimer's disease: Recommendations from the National Institute on Aging-Alzheimer's Association workgroups on diagnostic guidelines for Alzheimer's disease	ALZHEIMERS DEMENT
2	Mckhann GM	The diagnosis of dementia due to Alzheimer's disease: recommendations from the National Institute on Aging-Alzheimer's Association workgroups on diagnostic guidelines for Alzheimer's disease	ALZHEIMERS DEMENT
3	Livingston G	Dementia prevention, intervention, and care	LANCET
4	Sperling RA	Toward defining the preclinical stages of Alzheimer's disease: recommendations from the National Institute on Aging-Alzheimer's Association workgroups on diagnostic guidelines for Alzheimer's disease	ALZHEIMERS DEMENT
5	Jack CR	NIA-AA Research Framework: Toward a biological definition of Alzheimer's disease	ALZHEIMERS DEMENT

### Analysis of cited journal

Table 9 presented the top 5 co-cited journals. Neurology ranked first with 2703, implying it might be the most popular journal, followed by Journal of the American geriatrics society, Journal of Alzheimers disease, PLoS one and Alzheimers & Dementia. Only the Journal of Alzheimers disease had a centrality greater than 0.1 (centrality: 0.29). It’s worth noting that Lancet which ranked 9th had the highest impact factor (IF: 202.731).

**Table 9:** The top 10 co-cited Journal

Ranking	Times cited	Centrality	Year	Journal	IF (2021)
1	2703	0.05	2012	Neurology	12.258
2	2369	0.11	2012	Journal of the American geriatrics society	7.538
3	2008	0.02	2012	Journal of Alzheimers disease	4.16
4	1927	0.01	2012	PLoS One	3.752
5	1851	0.00	2012	Alzheimers & Dementia	16.655
6	1729	0.00	2012	Archives of neurology	7.419
7	1657	0.00	2012	Jama-journal of the American medical association	157.375
8	1611	0.03	2012	Neurobiology of aging	5.133
9	1605	0.00	2012	Lancet	202.731
10	1443	0.01	2012	International journal of geriatric psychiatry	3.85

## Discussion

In this study, 4713 publications from WoSCC and 304 publications from CNKI between the year of 2012–2022 were included for the comparatively bibliometric analysis on old adults’ CI, while the results showed that this research field was still young and the trend of articles published in WoS and CNKI were still on the rise.

Result from WoS showed that USA, University of California, Petersen RC were the most influential country, institute and scholar respectively; Folstein MF and Neurology was the most cited scholar and journal, and paper titled ‘The diagnosis of mild cognitive impairment due to Alzheimer's disease: Recommendations from the National Institute on Aging-Alzheimer's Association workgroups on diagnostic guidelines for Alzheimer's disease’ [18] was the most cited reference. The top 10 keywords were Alzheimer’s disease, mild cognitive impairment, dementia, risk, prevalence, elderly patient, impairment, decline, association, disease. Result from CNKI showed that Peking Union Medical College, Dan Liu were the most influential institution and scholar separately. The top 10 keywords were cognitive function, influencing factors, review, depression, risk factors, dementia, prevalence, fragility, nursing home and screening.

### Countries and institutions

In terms of countries analysis, the United States was the most productive country in this field, which had an overwhelmingly higher number of publications than other countries. Also notably, although the number of publications in China ranked second, the cooperation with other countries was low. As for Institutions, the six institutions were all from the United States, indicating United States still maintained the dominant position in this field. The data from CNKI showed that publication of Chinese institutions was comparatively low. However, the data both from WoS and CNKI showed almost all the institutions were engaged in independent research, and very few collaborations between different institutions were found (only Columbia University and University College London had a centrality more than 0.1). Therefore, more collaboration and communications between different institutions were encouraged on the research field of old adults’ CI.

### Authors, co-cited authors, cited reference and cited journal

It was found that many authors tend to form a relatively stable group of collaborators and has generated several major author clusters via the list of the top 10 most prolific authors, which could be a potential reason for the lower centrality. Simultaneously, as shown in Fig. 5(A), the authors with a higher volume of publications have published fewer articles in recent years. Data from CNKI showed the publications of all the Chinese authors were low, but consisted with WoS, a relatively stable group had formed and the centrality of all authors were low, only the 3 authors with the highest number of publications have a close collaboration. Petersen RC, Jack CR and Knopman DS had contributed the most publications and played important roles in the field. Petersen RC and Jack CR not only contributed the most publications, but also ranked second and third in terms of cited-authors, respectively (as shown in Table 10). Combined with Table 8, it could be seen that Petersen RC and Jack CR was also on the top 5 cited reference. All these indicated Petersen RC and Jack CR had published a large number of articles of high quality and were experts in the field. Folstein MF was the most cited author, who devised a simplified, scored form of the cognitive mental status examination, the “Mini-Mental State” (MMS). This examination shortened the response time from 30 min to 5–10 min by only asking cognitive aspects of mental functions [19]. The development of this examination brought him a lot of attention. However, the centrality of all these authors was less than 0.1, thus more collaborations are expected.

**Table 10:** Top 10 cited authors on old adults’ CI in WoS

Ranking	Cited author	Frequency	Ranking	Cited author	Centrality
1	Folstein MF	1063	1	Luchsinger JA	0.06
2	Petersen RC	888	2	Ganguli M	0.06
3	Jack CR	479	3	Hardy J	0.05
4	Morris JC	466	4	Fratiglioni L	0.05
5	Albert MS	350	5	Morris MC	0.05
6	Mckhann GM	317	6	Biessels GJ	0.05
7	Braak H	297	7	Mckhann GM	0.04
8	Nasreddine ZS	295	8	Wilson RS	0.04
9	Yesavage JA	286	9	Barnes DE	0.04
10	APA ^a^	283	10	Roberts RO	0.04

^a^ *APA* American Psychiatric Association

As shown in Table 8, Albert MS from Johns Hopkins University, whose paper titled “The diagnosis of mild cognitive impairment due to Alzheimer's disease: Recommendations from the National Institute on Aging-Alzheimer's Association workgroups on diagnostic guidelines for Alzheimer's disease” [18] was the most cited; Mckehann GM from Johns Hopkins University, whose paper titled “The diagnosis of dementia due to Alzheimer's disease: recommendations from the National Institute on Aging-Alzheimer's Association workgroups on diagnostic guidelines for Alzheimer's disease” [20] ranked 2rd, Sperling RA from Harvard Medical School, whose paper titled “Toward defining the preclinical stages of Alzheimer's disease: recommendations from the National Institute on Aging-Alzheimer's Association workgroups on diagnostic guidelines for Alzheimer's disease” [21] ranked fourth. It was worth noting three of the top five reference were all on the diagnosis guidelines on CI, and authors of these reference all came from top institutions and had written numerous articles with high quality.

Journal analysis could reflect the influential journals in the research field [22]. The impact factors of the top 10 journals ranged from 3 to 203, and two journals’ IF in 2021 over 100.00. In addition, as mentioned earlier, four of the top five reference were published in Alzheimers & Dementia, which ranked fifth in the cited journal. All these suggested that the studies were reliable and of high quality.

### Keywords, research hot spots and frontiers

Keywords and keyword clustering analysis in WoS and CNKI both could be classified into three main topics, which could be further used to show the research hot spots and frontiers on the old adults’ CI.

The first aspect of keywords was the related disease and symptoms of CI. Researches from WoS showed that CI ranges from mild to severe, and is one of the most common and disabling non-motor symptoms in elderly individuals. Mild cognitive impairment (MCI) is considered a preclinical transitional stage between healthy aging and dementia. Dementia includes Alzheimer's disease (AD), vascular dementia, frontotemporal dementia and so on; among them, AD is the main cause of dementia [23], the loss of independence is the primary feature differentiating dementia from mild cognitive impairment. An individual could transit from the asymptomatic phase to the symptomatic predementia phase [4], and from the symptomatic predementia phase to dementia onset. Consistent with Table 5, Alzheimer's disease, mild cognitive impairment and dementia were the top three keywords. Diagnostic guideline was an main cluster type, recommendations about diagnostic guidelines for CI were made by the National Institute on Aging-Alzheimer’s Association workgroups, including the characterization of the preclinical [21], MCI phases and dementia due to Alzheimer's disease [18]. As shown in Table 8, these articles that clarified diagnostic criteria were most frequently cited. CNKI focused mainly on the field of MCI [24, 25]. One study indicated that depression was the most common complication of MCI in the elderly, and it seriously affected the physical and mental health of the elderly when co-morbid with MCI [26]. Another article suggested that square dancing with Chinese characteristics could be an effective intervention to improve cognitive function and depression in elderly MCI patients [27]. Besides, the epidemiological status and intervention of dementia were also involved [28, 29], while AD in dementia was more often studied. As shown in Table 5, cognitive function, depression and dementia were the top 10 keywords.

The second aspect of keyword was the risk factors of CI, which was mentioned both the WoS and CNKI. In WoS, a cohort study indicated the risk factors and influencing factors of CI were associated with age, gender, place of residence, education level, disability, family annual income, smoking, sleeping duration, IADL impairment, depression, community activities participation [30]. A cross-sectional analysis showed preventing abdominal obesity was associated with better cognitive performance among both males and females [31]. Another cross-sectional analysis and narrative review both indicated participants with high blood pressure had significantly lower cognition compared to the others [32] and hypertension showed the strongest association with vascular dementia [33]. These finding were in line with WHO’s the first guidelines for reduction of risk of cognitive decline and dementia, which also acknowledge a number of factors that may contribute to cognitive impairment, such as physical activity, diet, overweight or obesity, tobacco and alcohol use, hypertension, and diabetes [34]. Besides, data from CNKI presented that empty nester, and discovered the prevalence of CI among them was higher than the general elderly, which was considered in relation to the lack of child care, infrequent communication with relatives and friends, reduced social activities, high prevalence of psychological disorders and chronic diseases [35]. Moreover, CNKI emphasized the impact of frailty since 2018, as shown in Fig. 5(C), which was a complex and multifaceted public health issue highly prevalent in older adults leading to cognitive frailty [36] and the risk of cognitive decline increases 3.6 times in frail older adults [37, 38].

The difference between WoS and CNKI existed in the third aspect of keyword. For WoS, the mechanism of cognitive decline had gained more attention, since 8 keywords of the 25 keywords with strongest citation bursts associated with mechanism, as shown in Fig. 4(C), such as Pittsburgh compound b, medial temporal lobe, hippocampal, atrophy, prefrontal cortex, genome wide association, hippocampal atrophy and oxidative stress. This indicated researches on the mechanisms of cognitive decline were once hot in the past few years, but it could also be seen keywords on the system, anxiety, medication use, s disease, virtual reality and oxidative stress are becoming more and more important. Researches on intervention had gained more attention in CNKI. A mean of intervention, non-pharmacological treatments had attracted extensive attention of scholars from CNKI and WoS. Progress was being made in developing pharmacological interventions specific for Alzheimer’s disease or dementia [34], however, one study showed that pharmacological interventions have not shown much efficacy in changing the course of AD dementia [39], effective drugs for cognitive impairment still need to be developed. Therefore, to ensure that people with dementia could maintain a level of functional ability, a more definitive cognitive assessment and effective nonpharmacological intervention became the most important task. On the one hand, virtual reality that could enhance therapeutic gains had drawn the attention from WoS [40]. On the other hand, the traditional Chinese exercise, Tai Chi with various form, gentle movements and moderate intensity was presented as treatment of CI [41–43]. The research form CNKI emphasized more on screening, which was an important tool adopted and clinical treatment channels to promote early detection, early intervention, early diagnosis and early treatment [44].

### Limitations

There are some limitations for this study [45]. Firstly, due to the limitation of Citespace software, this study only selected data sources from the WoSCC database and published in English. Secondly, co-cited authors, cited reference and cited journal were not able to be included for analysis for CNKI database when using Citespace, implying the data may not be comprehensive enough and the analysis may be incomplete.

## Conclusions

Articles published on old adults’ CI were drawing an increasing amount of attention from 2012 to 2022 both in WoS and CNKI. Keywords of CI in WoS and CNKI both focused on risk factors, related disease and symptom, yet WoS contributed more to the mechanism and CNKI contributed more to the intervention.

## Data Availability

Not applicable.

## Abbreviations

CI: Cognitive impairment
WoS: Web of Science
CNKI: China National Knowledge Infrastructure
AD: Alzheimer's disease Declarations
