# Correction: A comparative study of bibliometric analysis on old adults’ cognitive impairment based on web of science and CNKI via CiteSpace

**DOI:** 10.1186/s13561-024-00483-w

**Published:** 2024-02-22

**Authors:** Shuyi Yan, Mingli Pang, Jieru Wang, Rui Chen, Hui Liu, Xixing Xu, Bingsong Li, Qinling Li, Fanlei Kong

**Affiliations:** 1 Centre for Health Management and Policy Research, School of Public Health, Cheeloo College of Medicine, Shandong University, Jinan, 250012 China; 2 NHC Key Lab of Health Economics and Policy Research, Shandong University, Jinan, 250012 China; 3 Institute of Health and Elderly Care, Shandong University, Jinan, China

**Correction: Health Econ Rev 13, 56 (2023)**

10.1186/s13561-023-00470-7

Following publication of the original article [1], the missing co-author Qinling Li was added as the eighth author. Dr. Li’s name was inadvertently removed during the production process. Dr. Li’s affiliations are the following:

^1^Centre for Health Management and Policy Research, School of Public Health, Cheeloo College of Medicine, Shandong University, Jinan 250,012, China.

^2^NHC Key Lab of Health Economics and Policy Research, Shandong University, Jinan 250,012, China.

^3^Institute of Health and Elderly Care, Shandong University, Jinan, China.

Moreover, the text “Present Adress” was removed from affiliation 3.

The author group has been updated above and the original article [1] has been corrected.
